# Palm kernel meal regulates the expression of genes involved in the amino acid metabolism in the liver of Tibetan sheep

**DOI:** 10.1186/s12917-024-04193-7

**Published:** 2024-07-23

**Authors:** Boyan Ma, Fengshuo Zhang, Sayed Haidar Abbas Raza, Zhenling Wu, Quyangangmao Su, Yu Zhang, Zhiyou Wang, Tahani Ahmad ALMatrafi, Bandar Hamad Aloufi, Heba I. Ghamry, Mustafa Shukry, Shengzhen Hou, Linsheng Gui

**Affiliations:** 1 College of Agriculture and Animal Husbandry, Qinghai University, Xining, 810016 Qinghai Province People’s Republic of China; 2 Guangdong Provincial Key Laboratory of Food Quality and Safety, South China Agricultural University, Guangzhou, China; 3 Guangdong Provincial Key Laboratory of Utilization and Conservation of Food and Medicinal Resources in Northern Region, Shaoguan University, Shaoguan, 512005 China; 4 College of Animal Science and Technology, Northwest A&F University, Yangling, 712100 Shaanxi China; 5 Anatomy Department, College of Medicine, King Saud University, Riyadh, Saudi Arabia; 6 Biology Department, Faculty of Science, University of Ha’il, Ha’il, Saudi Arabia; 7 Nutrition and Food Science, Department of Biology, College of Science, King Khalid University, P.O. Box 960, Abha, 61421 Saudi Arabia; 8 Department of Physiology, Faculty of Veterinary Medicine, Kafrelsheikh University, Kafrelsheikh, 33516 Egypt

**Keywords:** Palm kernel meal, Transcriptome, Liver, Nutrient, Tibetan sheep

## Abstract

**Background:**

Palm kernel meal (PKM) is a by-product of oil palm kernel after oil extraction, which is widely used in animal feeds due to its high energy content. This study aimed to investigate the impact of supplementing Tibetan sheep with PKM on their hepatic phenotype, oxidative stress and immune response. A total of 120 Tibetan lambs (Initial weight = 12.37 ± 0.92 kg) were randomly assigned into four groups: control group (C group, 0% PKM diet), low group (L group, 15% PKM diet), middle group (M group, 18% PKM diet), and high group (H group, 21% PKM diet) on a dry matter basis. The feeding experiment was performed for 130 d, including a 10 d adaption period.

**Results:**

Results showed that the level of GSH-Px were higher in the H and M groups than in the C and L groups (*P* < 0.05). The levels of IgM and TNF-α were higher in the M group when compared to those on the C group (*P* < 0.05). The level of IgA was significantly higher in the M group than in the H group (*P* < 0.05). Additionally, compared with the others groups, the hepatocytes in the M group displayed a radial arrangement, forming hepatic plates that were centered around the central vein. The transcriptome results revealed that proteasome 26 S subunit, ATPase 3 (*PSMC3*), proteasome 26 S subunit, ATPase 5 (*PSMC5*), proteasome 26 S subunit ubiquitin receptor, non-ATPase 4 (*PSMD4*), proteasome activator subunit 1 (*PSME1*), acyl-CoA dehydrogenase short/branched chain (*ACADSB*), enoyl-CoA hydratase, short chain 1 (*ECHS1*), serine dehydratase (*SDS*), ornithine transcarbamylase (*OTC*), and phenylalanine hydroxylase (*PAH*) were the hub genes regulating the amino acid metabolism in the liver.

**Conclusions:**

In summary, dietary 18% PMK supplementation contributed to improve the hepatic phenotype, oxidative stress and immune response through regulating the expression of related genes.

## Introduction

The rapid development of the livestock industry has caused an unprecedented increase in the demand for livestock resources [1]. To alleviate the competition for food between humans and animals, it is necessary to explore alternative feedstuffs, with the development and utilization of agricultural by-product waste attracting increasing attention [2]. By-products of agro-processing, a special form of agricultural resources, are widely used in various industries such as pig, laying hen, and cattle [3].

Among many agricultural by-products, palm kernel meal (PKM) has several advantages, including being abundant, cheap [4], rich in nutrition [5], abundant in vitamin E [6], and has antioxidation properties, among others. Thus, it has the potential to be used as an animal feed. Wyngaard [7] et al. found that adding 400 g/kg PKC to the grazing dairy cows’ concentrate had no effect on milk yield, fat, or protein content. Ribeiro [8] et al. showed that the addition of 19.5% palm kernel to lamb rations had no significant effect on the quality of lamb meat. At present, studies on palm kernel meal are all based on its digestibility, but animal utilization of key palm kernel meal nutrients is not well understood.

Based on studies concerning livestock, we hypothesized that including PMK in the diet might influence hepatic function in Tibetan sheep. Thus, the objective of this study was to investigate the impact of dietary PKM supplementation on hepatic phenotype, oxidative stress, and immune response in the livers of Tibetan sheep using RNA-Seq sequencing technology.

## Materials and methods

### Animal diet and sample collection

One hundred and twenty healthy Tibetan lambs with an initial mean weight of 12.37 ± 0.92 kg (2 months old) were randomly selected from a commercial Tibetan sheep farm (Gonghe, Qinghai Province, China). According to dietary treatments, the lambs were randomly divided into four treatments, including a control group (C group, 0% PKM diet), low group (L group, 15% PKM diet), middle group (M group, 18% PKM diet), and high group (H group, 21% PKM diet) on a dry matter basis. The dietary concentrates of the four treatments contained 30% roughage (oat silage and oat hay to dry matter ratio of 1:1) and 70% concentrate. After a 10-day adaption phase, the Tibetan lambs were subjected to a 120-day formal trial having *ad* libitum access to food and water. The composition of the basic diet and nutrient levels is shown in Table 1.

**Table 1:** Concentrate supplement and nutrient levels in diet (dry matter basis %)

Ingredient	C group	L group	M group	H group
Ingredients				
Oaten hay	15.00	15.00	15.00	15.00
Oats silage	15.00	15.00	15.00	15.00
Corn	45.85	37.94	36.26	34.65
Soybean meal	5.60	3.50	3.50	2.80
Rapeseed meal	11.20	10.50	10.50	10.50
Cottonseed meal	2.80	3.01	2.59	2.80
Palm kernel meal	0.00	10.50	12.60	14.70
NaCl	0.35	0.35	0.35	0.35
Limestone	0.70	0.70	0.70	0.70
Premix^1)^	3.50	3.50	3.50	3.50
Chemical composition				
Gross energy (MJ/kg)	10.46	10.49	10.53	10.67
Crude protein	10.62	10.60	10.61	10.63
Ether extract	1.89	2.40	2.51	2.61
Crude fiber	2.79	4.16	4.43	4.72
Neutral detergent fiber (NDF)	21.29	25.62	26.44	27.33
Acid detergent fiber (ADF)	12.87	15.22	15.67	16.15
Ash	1.96	1.69	1.64	1.59
Calcium	0.37	0.36	0.36	0.36
Phosphorus	0.30	0.26	0.25	0.25

^1)^ The premixes provided Fe 45.0 g, Cu 6.0 g, Mn 30.0 g, Zn 50.0 g, iodine 500.0 mg, Se 125.0 mg, Co 125.0 mg, VA12,000 IU, VD 2 400 IU, and VE 240 IU per Kg of diet. The nutrient levels were measured

At the end of the experiment, three Tibetan sheep in each treatment were randomly selected for slaughter in a commercial slaughterhouses. The liver tissue were collected and placed into liquid nitrogen for RNA extraction, while the remaining tissue samples were fixed in 4% paraformaldehyde for tissue- sectioning.

### Antioxidant and immune response index assays

The immune response and antioxidant indices were measured using Enzyme-linked immunosorbent assay (ELISA). The immune response indicators included interleukin-1β (IL-1β), tumor necrosis factor-α (TNF-α), immunoglobulin A (IgA), IgG, and IgM, while the antioxidant indicators included superoxide dismutase (SOD), total antioxidant capacity (T-AOC), and glutathione peroxidase (GSH-Px) levels. The ELISA kits used in this study were procured from Jiancheng Bioengineering Institute (Nanjing, China).

### Histological analysis of the liver

The collected liver samples were dehydrated, trimmed, dipped in wax, embedded, sectioned, and then stained with hematoxylin-eosin (H&E). The randomly selected morphologically intact target areas of liver were observed by digital microscope (DP2-BSW, Olympus Corporation, Tokyo, Japan) at 400× magnification. The cross-sectional areas of the liver were determined using Image-Pro Plus software version 6.0 (Media Cybernetics Inc, Bethesda, MD, USA).

### Transcriptome sequencing

Total RNA was extracted using the Trizol reagent (Thermo Fisher Scientific, Waltham, MA, USA) following the manufacturer’s protocol. The total RNA quantity and purity were analyzed using the Bioanalyzer 2100 (Agilent, CA, USA) and RNA 6000 Nano Lab Chip Kit (Agilent, CA, USA). High-quality RNA samples with RIN number > 7 were used to construct the sequencing library (GENE DENOVO, Guangzhou, China). Thereafter, 2 × 150 bp paired-ends (PE150) were sequenced on the Illumina Novaseq™ 6000 platform (LC-Bio Technology, Hangzhou, China) following the manufacturer’s protocol. Reads obtained from the sequencing machines includes raw reads containing adapters or low quality bases which will affect the following assembly and analysis. Thus, to get high quality clean reads, reads were further filtered by fastp (version 0.18.0). The reads containing adapters and more than 10% of unknown nucleotides (N) were removed. Then, low quality reads containing more than 50% of low quality (Q-value ≤ 20) bases were removed. Clean reads were then mapped to reference genome (Genome: Oar_Version 3.1) using HISAT2 (https://daehwankimlab.github.io/hisat2/, version hisat2-2.0.4).

### Analysis of gene expression

The expression of genes were estimated using string Tie software (http://ccb.jhu.edu/software/stringtie/, version: stringtie-1.3.4d) and ballgown (http://www.bioconductor.org). Here, the expression levels of all transcripts were estimated, and the expression abundance of mRNAs was performed by calculating fragment per kilobase of transcript per million mapped reads (FPKM) value. Principal component analysis (PCA) was performed using the princomp function in the R software (http://www.r-project.org/). Analysis for differential gene expression between different groups (and by edgeR between two samples) was performed using DESeq2 software. The threshold for differential gene expression was set at false discovery rate (FDR) ˂ 0.05 and absolute fold change ˃ 2.

### Short time-series expression miner (STEM) analysis

The clustering of the differently expressed genes (DEGs) in the liver tissue of Tibetan sheep in different PKC groups was performed using the STEM software (version 1.3.11). Each gene was assigned to the closest profile using the Pearson correlation-based distance metric. A permutation-based test was used to quantify the expected number of genes that would be assigned to each profile to determine the significance level of a given transcriptome profile [9]. Profiles with a *P* value ˂ 0.05 were considered significantly enriched. Then, Kyoto Encyclopedia of Genes and Genomes (KEGG) and Reactom Pathway Database were applied to identify the pathways regulated by the DEGs. In order to identify the core differential genes, the enriched genes were entered into the STRING database (version 9.1), and the Cystoscape plug-in cytoHubba was used to detect the hub genes with the maximal clique centrality algorithm.

## Results

### Antioxidation activities and immune response

The effects of the experimental treatments on antioxidation activities and immune response were showed in Table 2. The level of GSH-Px were higher in the H and M groups than in the C and L groups (*P* < 0.05). No significant difference in the levels of T-AOC and SOD among 4 groups were observed (*P* > 0.05). Additionally, the levels of IgM and TNF-α were higher in the M group when compared to those on the C group (*P* < 0.05). The level of IgA was significantly higher in the M group than in the H group (*P* < 0.05).

**Table 2:** Antioxidant and immune response index

Items	Groups				*P* value
	C group	L group	M group	H group	
GSH-Px (pmol/mL)	2.28 ± 0.01 ^b^	2.27 ± 0.03 ^b^	2.34 ± 0.01 ^a^	2.39 ± 0.02 ^a^	0.01*
T- AOC (µ/mL)	1.25 ± 0.04	1.19 ± 0.04	1.32 ± 0.04	1.26 ± 0.07	0.36
SOD (pg/mL)	2.07 ± 0.09	2.03 ± 0.09	2.27 ± 0.11	2.00 ± 0.06	0.16
IgA (µg/mL)	1.49 ± 0.01 ^ab^	1.48 ± 0.01 ^ab^	1.51 ± 0.01 ^a^	1.46 ± 0.01 ^a^	0.01*
IgG (µg/mL)	1.15 ± 0.01	1.16 ± 0.01	1.16 ± 0.02	1.18 ± 0.01	0.39
IgM (µg/mL)	0.51 ± 0.04 ^b^	0.63 ± 0.04 ^a^	0.66 ± 0.03 ^a^	0.65 ± 0.03 ^a^	0.02*
IL-1β (ng/L)	1.50 ± 0.01	1.50 ± 0.01	1.50 ± 0.01	1.51 ± 0.01	0.75
TNF-α (ng/L)	1.31 ± 0.01 ^b^	1.34 ± 0.01 ^ab^	1.39 ± 0.01 ^a^	1.35 ± 0.01 ^ab^	0.01*

*Note* Data in the same line with the same or no lowercase letters indicate nonsignificant differences (*p* > 0.05), and data with different lowercase letters indicate significant differences (*p* < 0.05). * represent to significant difference (*P < 0.05*)

### Histological observation

**Fig. 1:**
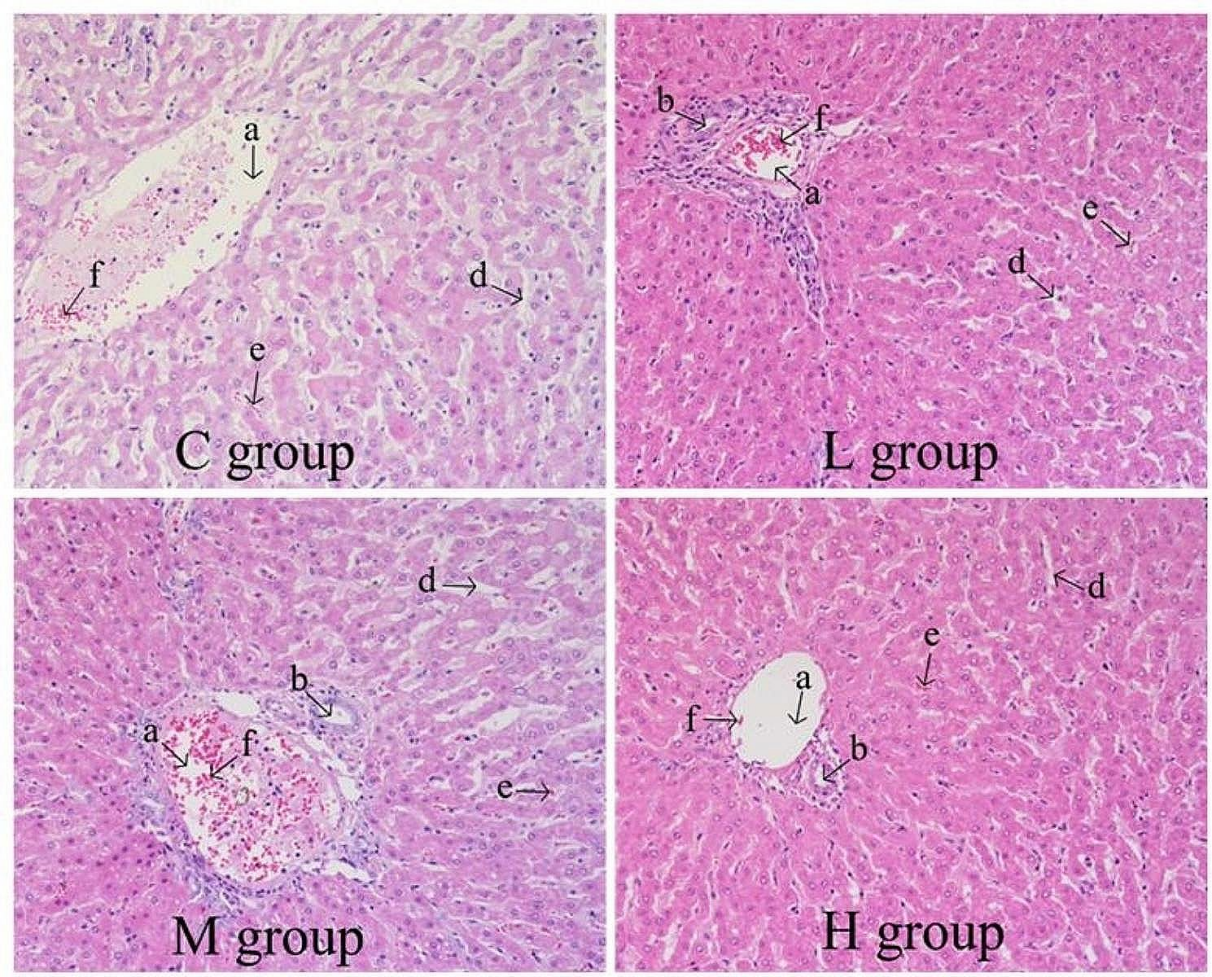
Effect of varying supplement levels of PMK on hepatic phenotypes. C group was supplement of 0% PKM. L group was supplement of 15% PKM. M group was supplement of 18% PKM. H group was supplement of 21% PKM. HE staining, 400x. a: Interlobular veins, b: Interlobular artery, d: Sinus Periphery Gap, e: Hepatic Blood Sinusoids, f: Macrophages

As illustrated in Fig. 1, varying supplement levels of PMK resulted in obvious changes in liver histomorphology. Compared with the others groups, the hepatocytes in the M group displayed a radial arrangement, forming hepatic plates that were centered around the central vein. Additionally, these plates anastomosed with each other, forming a lost-like structure.

### Transcriptome sequencing

The twelve complementary cDNA libraries produced a combined 4.94 million raw reads. After removing low-quality reads, 4.56 million high-quality clean reads were obtained from the twelve cDNA libraries. Sequences with Q30 ranged from 98.09 to 98.53%. The mapping rate for reads was ˃90%, with an average of 99.09% (Table 3), demonstrating the high quality of the reads and their suitability for further analyses.

**Table 3:** Basic sequencing date statistics for each sample

Sample	Raw Data	Valid Data	Valid Ration %	Q30%	Mapped reads	Mapped Ration %
C_1	46,717,438	44,478,210	95.21	98.44	42,079,029	94.61
C_2	40,958,038	38,320,448	93.56	98.53	36,734,188	95.86
C_3	41,135,596	37,742,944	91.75	98.31	35,107,506	93.02
L_1	40,464,468	37,207,482	91.95	98.28	35,136,192	94.43
L_2	42,533,922	39,699,818	93.34	98.22	36,494,158	91.93
L_3	43,553,722	41,387,304	95.03	98.22	38,132,407	92.14
M_1	37,435,188	33,808,186	90.31	98.16	31,999,077	94.65
M_2	38,073,870	33,937,550	89.14	98.37	31,967,332	94.19
M_3	39,028,768	35,530,122	91.04	98.18	33,426,242	94.08
H_1	40,495,710	37,870,604	93.52	98.29	35,460,517	93.64
H_2	46,208,246	42,244,494	91.42	98.09	38,678,839	91.56
H_3	37,002,270	33,447,966	90.39	98.50	31,985,225	95.63

### Differentially expressed genes

**Fig. 2:**
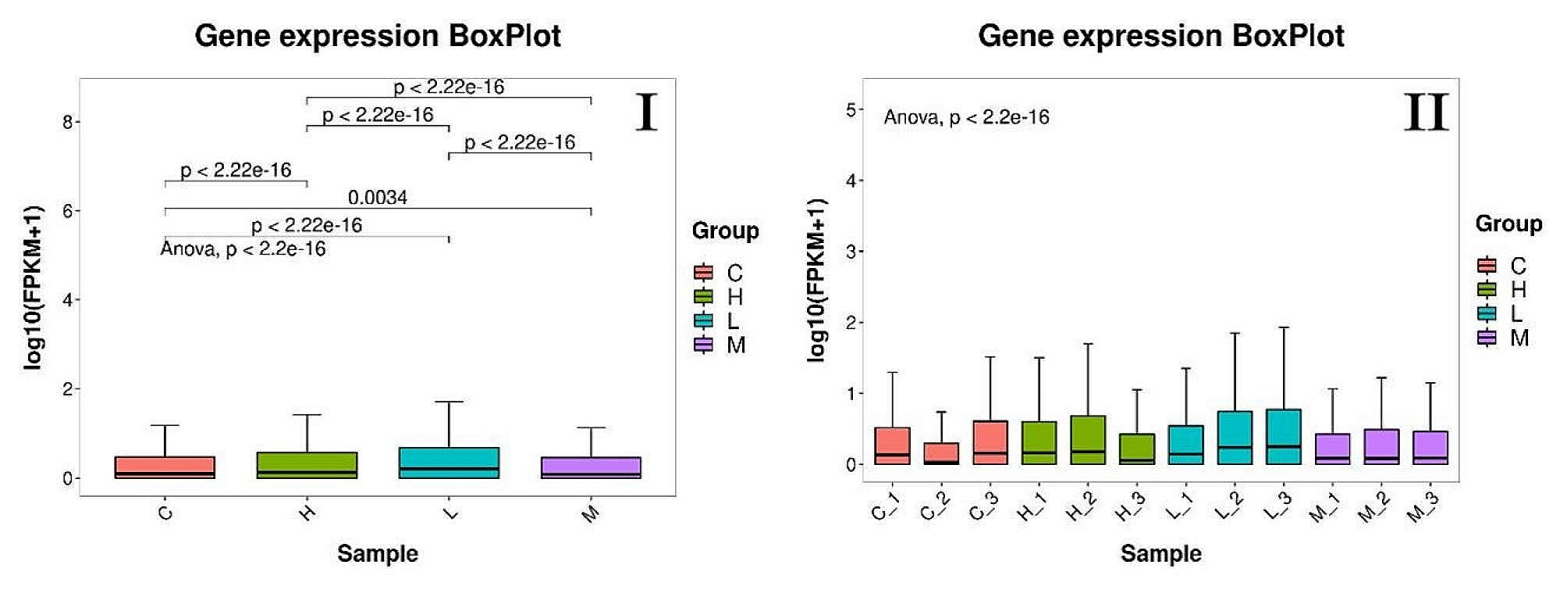
Gene expression BoxPlot. The horizontal coordinate statistic was derived based on the gene FPKM value. C group was supplement of 0% PKM. L group was supplement of 15% PKM. M group was supplement of 18% PKM. H group was supplement of 21% PKM. *P* < 0.05 indicates highly significant difference. **I**: Intergroup gene expression BoxPlot, to analyze the differences between groups. **II**: Gene expression within group, to analyze within-group uniformities

**Fig. 3:**
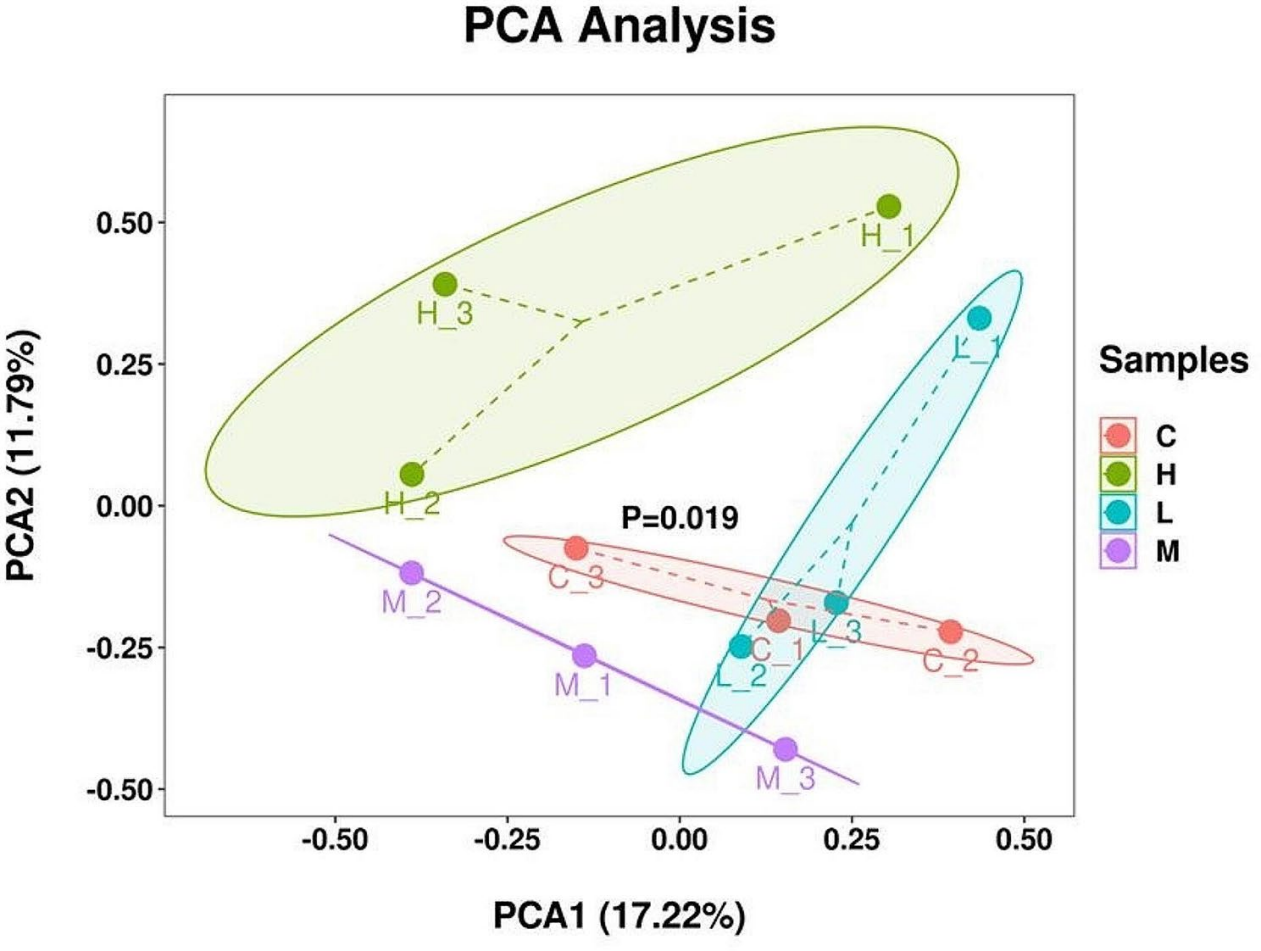
PCA Analysis. C group was supplement of 0% PKM. L group was supplement of 15% PKM. M group was supplement of 18% PKM. H group was supplement of 21% PKM. *P* < 0.05 indicates highly significant difference

We analyzed the gene expression of each sample based on the gene FPKM values. The results showed that the gene expression varied significantly among the groups, and the gene expression of the three parallel samples within the groups was always replicable (Fig. 2). PCA analysis of all the genes revealed that genes for samples in the same group clustered together, but samples for different groups clustered in different groups (Fig. 3). These results indicated that the expression of genes in samples in the same groups was homogeneous but heterogeneous for samples in different groups.

**Fig. 4:**
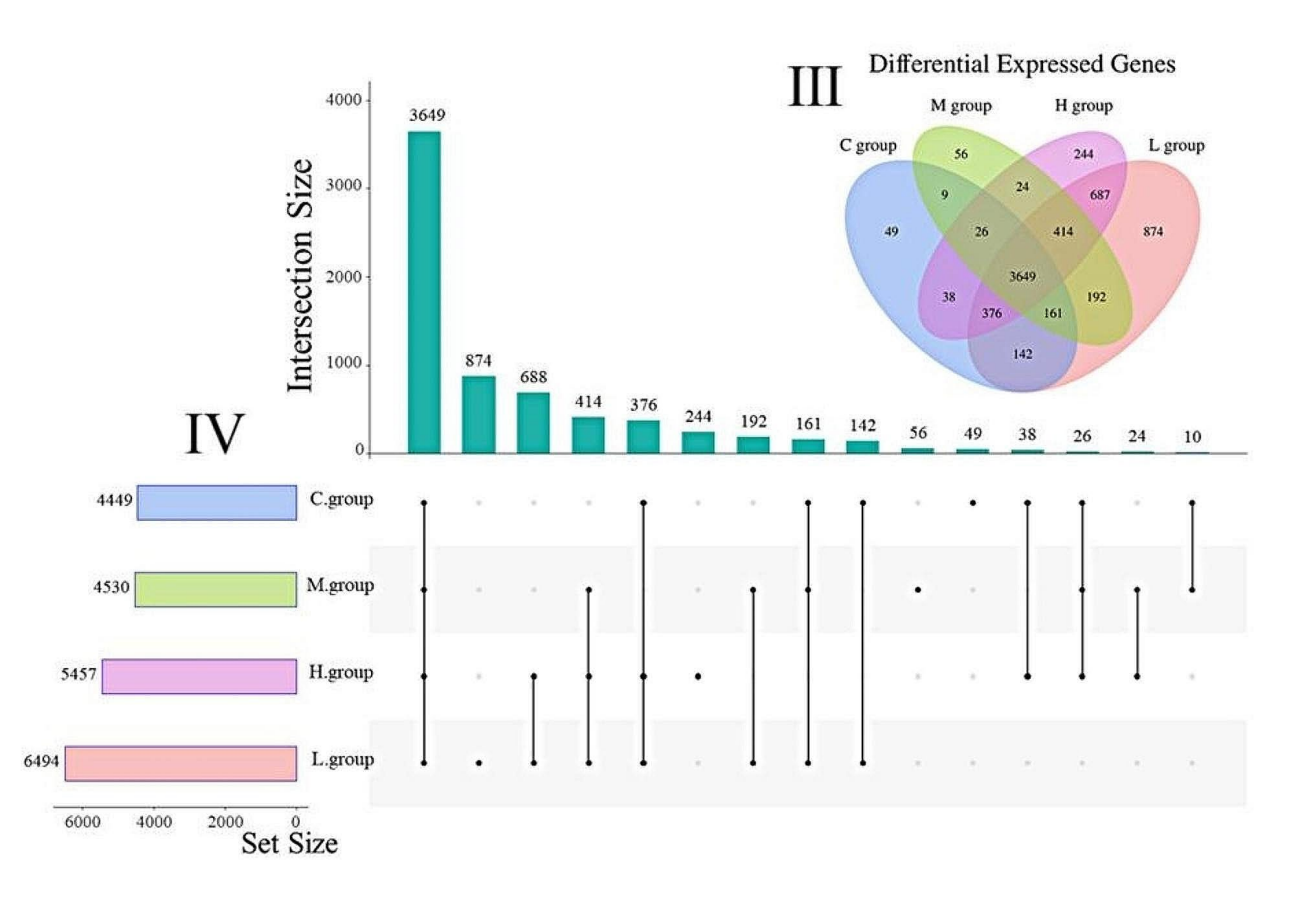
Differential Expressed Genes. Vertical bars shows the number of intersections, lines between points represents specific intersecting groups, and horizontal bars represents the amount of raw data for individual groups. C group was supplement of 0% PKM. L group was supplement of 15% PKM. M group was supplement of 18% PKM. H group was supplement of 21% PKM. III: Differential Expression Genes Venn Diagram, the overlap is co-annotated to the gene. IV: Gene expression UpSet Diagram, The black dots represent the samples, the line connecting the dots represents the sample intersection portion, the vertical bars represent the number of intersections, and the horizontal bars represent the sample raw volume

After excluding the differentially expressed genes with expressions lower than 1000 FPKM, the remaining differentially expressed genes were analyzed by Upset. The results showed that the 3649 genes shared by the four experimental groups, with 49 genes being unique to group C, 874 genes being unique to group L, 56 genes being unique to group M, and 244 genes being unique to group H (Fig. 4). The results indicated that PKM supplementation in feeds dysregulated the expression of several genes.

### STEM analysis

**Fig. 5:**
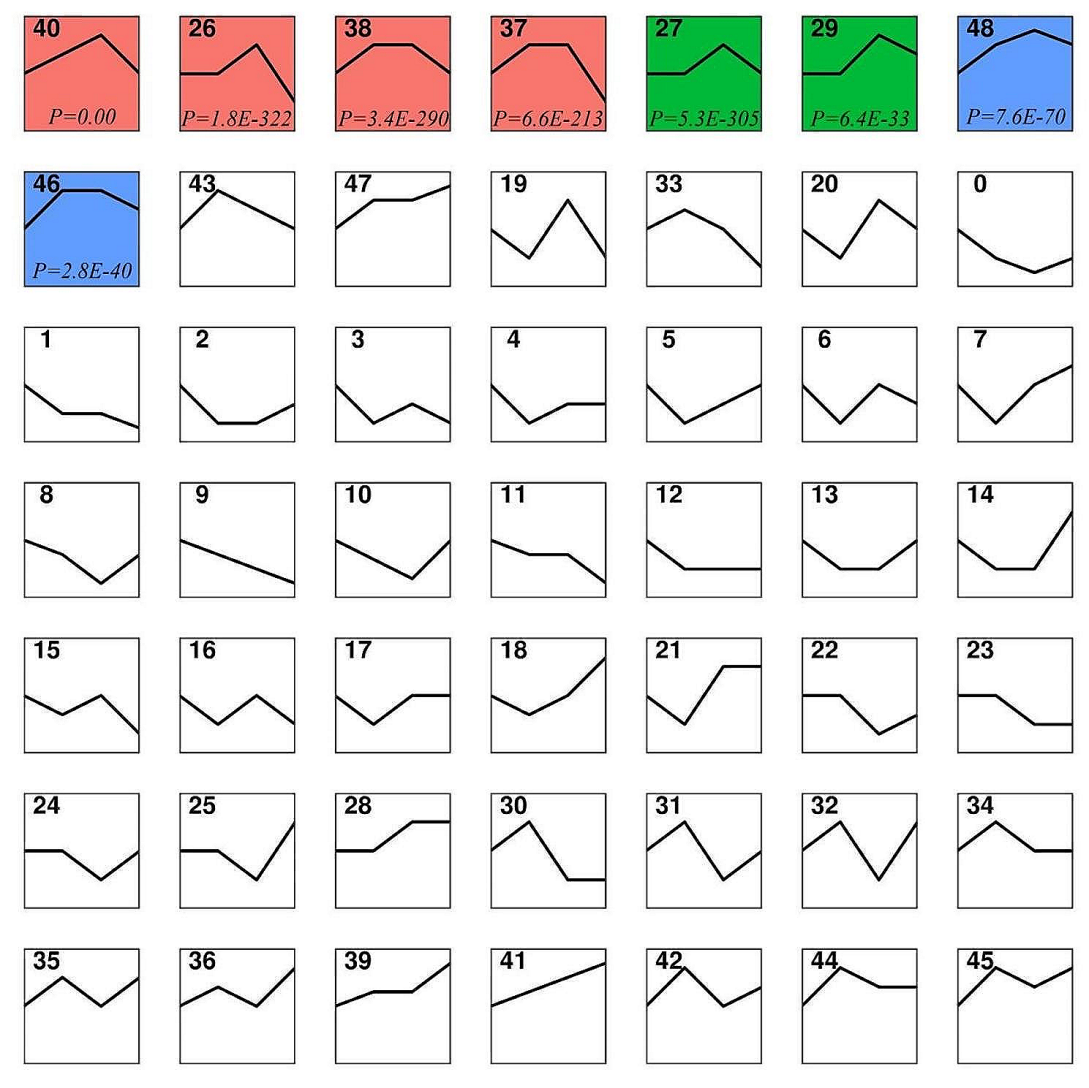
Short Time-Series Expression Miner (STEM) Analysis. A box represents a class of gene sets, a colored box represents a gene set with a significant trend, and the same color represents the same expression trend. C group was supplement of 0% PKM. L group was supplement of 15% PKM. M group was supplement of 18% PKM. H group was supplement of 21% PKM.

**Fig. 6:**
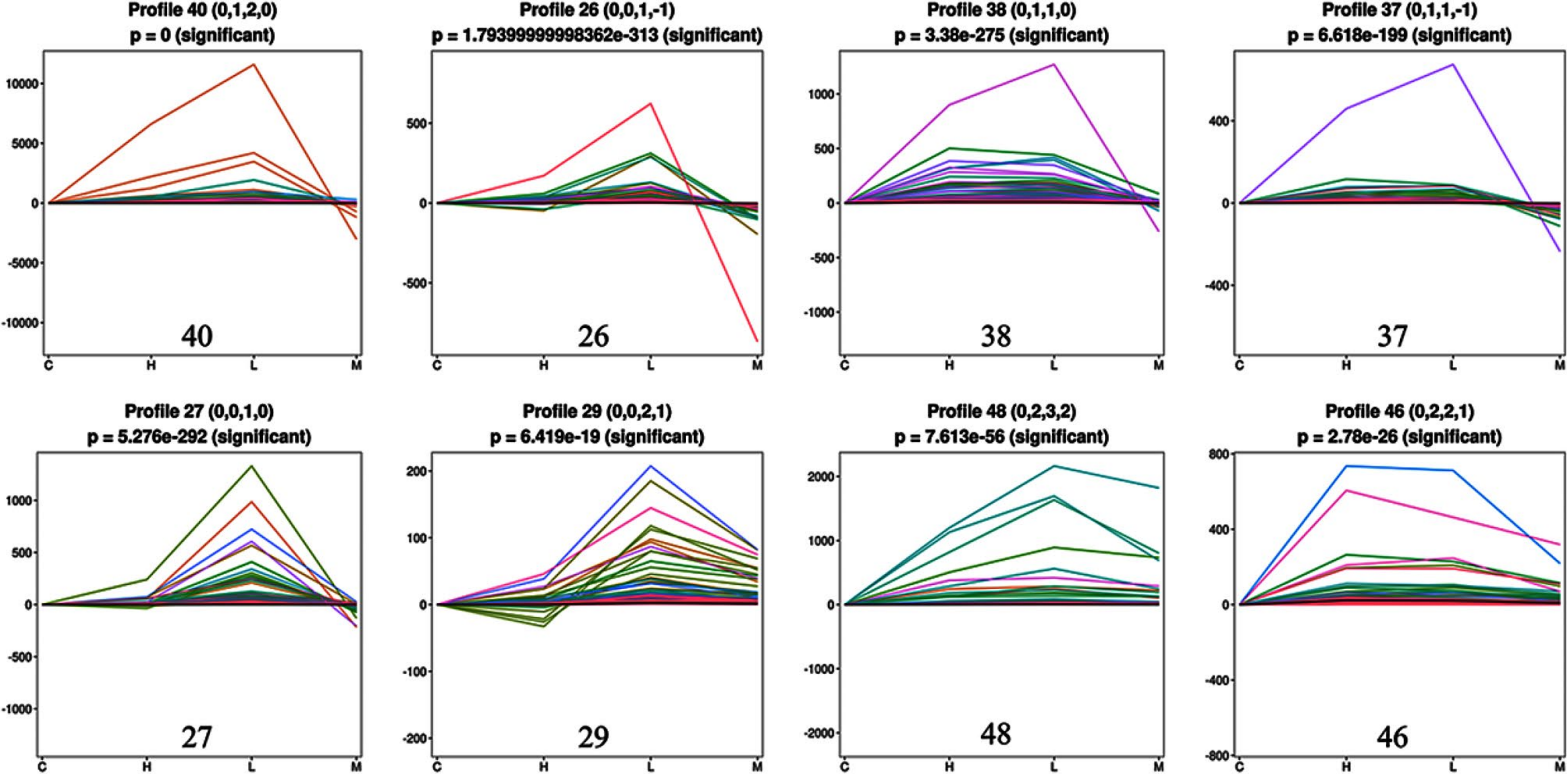
Gene sets with significant trends in STEM analysis. The positions of the dots represents a sample, the lines represents gene changes from the previous sample to the subsequent sample, and the lines of different colors represents different genes. C group was supplement of 0% PKM. L group was supplement of 15% PKM. M group was supplement of 18% PKM. H group was supplement of 21% PKM. *P* < 0.05 indicates highly significant difference

The expression profiles of 3649 DEGs were determined using cluster analysis based on STEM to obtain their expression patterns in the liver tissue of Tibetan sheep fed different PKM ratios. Forty-nine candidate genes were identified (Fig. 5), and the expression of eight of them was significantly dysregulated (*P* ˂ 0.05, Fig. 6). The eight gene profiles with significant enrichment were clustered into three groups. The first-group comprised four profiles: 40, 26, 38, and 37 (pink). The second-group consisted of two profiles, 27 and 29 (green), while 48 and 49 profiles were present in the third-group (blue).

### KEGG analysis

**Fig. 7:**
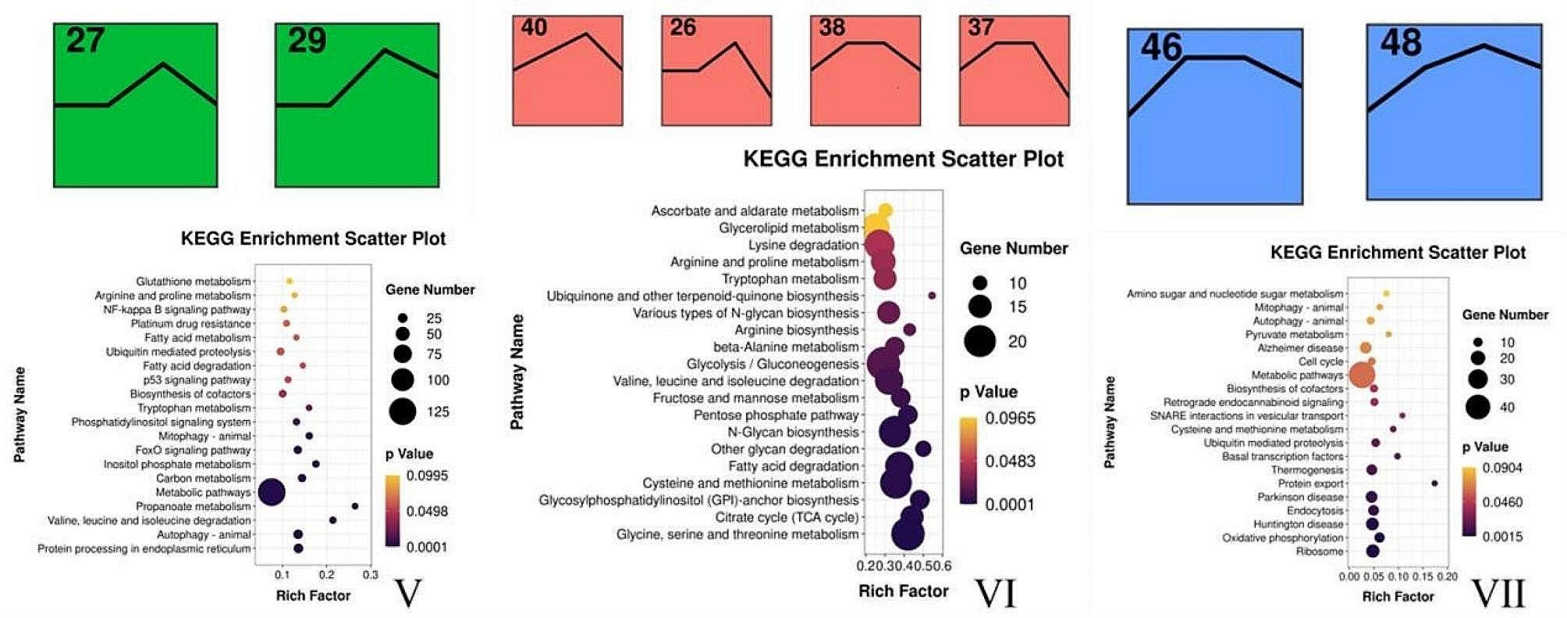
KEGG Enrichment Analysis. The size of the circle represents the number of genes enriched to the pathway, the larger the circle represents the more number of genes, and the color represents the significance, the deeper the color represents the lesser *P* value. *P* < 0.05 indicates significant difference. V: metabolic pathways enriched on 27 and 29 profile, VI: metabolic pathways enriched on 40, 26, 38 and 37 profile, VII: metabolic pathways enriched on 46 and 48 profile

**Fig. 8:**
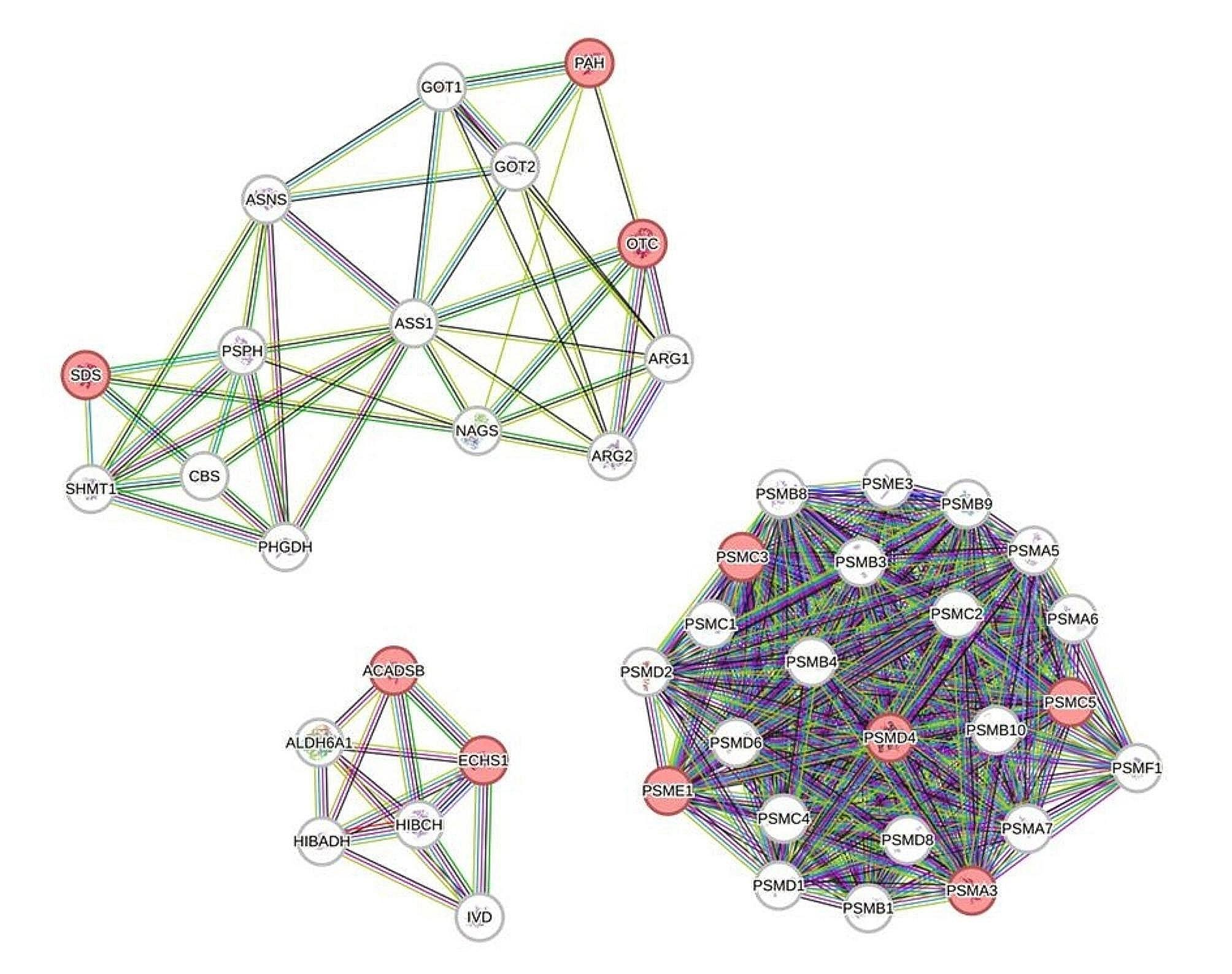
Protein-protein interaction (PPI) network. The figure is based on the Metabolism of amino acids and derivatives pathway in Reactom. where the pink represent hub genes regulated in liver tissue. *PSMC3*,*PSMC5*,* PSMD4*,* PSME1*,* PSMA3*,* ACADSB*,* ECHS1*,* SDS*,* OTC*,* PAH.*

The KEGG pathway enrichment analysis was performed based on the three groups clustered out by STEM analysis, and found that the enrichment to metabolic pathways in the first-group was mainly Glycolysis/Gluconeogenesis, Glycine, Serine, and Threonine metabolism, Cysteine, and Methionine metabolism ; the second-group was mainly enriched to Protein processing in the endoplasmic reticulum, Autophagy-animal, and Carbon metabolism pathway; and the third-group was mainly enriched to Ribosome, Oxidative Phosphorylation, and Endocytosis pathway (Fig. 7). The genes in the top three Pathways of the three groups were analyzed using the Reactom Pathway Database, and the results showed that the top Pathway was involved in the Metabolism of amino acids and their derivatives (Table 4). PPI analysis further revealed that *PSMC3*, *PSMC5*, *PSMD4*, *PSME1*, *ACADSB*, *ECHS1*, *SDS*, *OTC*, and *PAH* were the hub genes regulating the amino acid metabolism in the liver (Fig. 8).

**Table 4:** Reactom Pathway

Term ID	Term description	Observed gene count	Background gene count	Strength	False discovery rate
MAP-71,291	Metabolism of amino acids and derivatives	44	342	1.15	1.41E-32
MAP-9,755,511	KEAP1-NFE2L2 pathway	30	97	1.53	1.90E-31
MAP-8,939,902	Regulation of RUNX2 expression and activity	27	68	1.64	1.11E-30
MAP-5,358,346	Hedgehog ligand biogenesis	28	81	1.58	1.15E-30
MAP-5,610,785	GLI3 is processed to GLI3R by the proteasome	27	69	1.63	1.15E-30
MAP-9,759,194	Nuclear events mediated by NFE2L2	27	69	1.63	1.15E-30
MAP-9,762,114	GSK3B and BTRC: CUL1-mediated-degradation of NFE2L2	26	63	1.66	3.42E-30
MAP-983,169	Class I MHC mediated antigen processing & presentation	48	536	0.99	4.00E-30
MAP-5,607,761	Dectin-1 mediated noncanonical NF-kB signaling	26	65	1.64	5.16E-30
MAP-5,610,780	Degradation of GLI1 by the proteasome	26	65	1.64	5.16E-30
MAP-5,676,590	NIK–> noncanonical NF-kB signaling	26	65	1.64	5.16E-30
MAP-8,854,050	FBXL7 down-regulates AURKA during mitotic entry and in early mitosis	26	65	1.64	5.16E-30
MAP-1,169,091	Activation of NF-kappaB in B cells	26	73	1.59	4.29E-29
MAP-450,408	AUF1 (hnRNP D0) binds and destabilizes mRNA	25	62	1.65	4.60E-29
MAP-1,234,176	Oxygen-dependent proline hydroxylation of Hypoxia-inducible Factor Alpha	26	75	1.58	6.66E-29

## Discussion

Palm kernel meal is rich in micronutrients such as vitamins E and B [10]. Vitamin E is a fat-soluble vitamin that protects the liver from oxidative stress by reducing the production of free radicals and preventing damage to cell membranes [11]. Our results showed that the liver antioxidant index GSH-Px was significantly higher in the 18% and 21% PKM diets than in the other two groups. Mariana et al. (2023) found that PKM presented bioaccessible compounds after digestion with antioxidant activity [12]. Therefore, with increasing additions of PKM to the diet, an increase in the antioxidant bioaccessible compounds was also increased, thereby reducing the production of free radicals in the organism. Hepatocytes express innate immune receptors and present antigens to T cells to induce anti-inflammation.

PKM contains a mass of anti-nutritional factors, such as non-starch polysaccharides, trypsin inhibitors, etc. Studies have shown that a specific level of non-starch polysaccharides promoted the immune response, however, excessive supplement of PKM may result in the occurrence of disease [13]. Our analysis showed that IgA and IgM were significantly higher in the 18% PKM diets, whereas reduced when fed the 21% PKM. Huang et al. (2024) showed that the replacement of wheat bran with PKM will promoted intestinal immunity [14]. One possible explanation is that, dietary 21% PKM supplementation produced more accumulation of anti-nutrient, resulting in damage of immune system. Additionally, Our results showed that the TNF-α concentration was significantly higher in the 18% PKM supplement than in the other three groups. TNF is a proliferative factor secreted by the macrophages, which stimulated cyclic AMP (cAMP) generation, proliferation, and Ig production in B cells [15]. Overall, we comprehensively analyzed that dietary 18% PKM supplementation exhibited a better oxygenation and immunization in the liver.

As a critical hub for numerous physiological processes, the liver participated in the nutrient metabolism, immune system and intestinal homeostasis [16]. The functional structural unit of liver was hepatic lobule, the hepatic lobule will consist hepatocyte chords around the central vein. The hepatic chords are not tightly connected to each other, but appear as a net-like interstice, oxygen-rich blood from the hepatic artery mixes with nutrient-rich blood from the portal circulation in the sinusoid before flowing over the cells of the lobule and draining into the central vein [17]. Therefore, the interstitial space of the hepatic chords can also indirectly reflect the nutrient exchange. In our results, the 18% PKM supplementation had a larger hepatic chord interstitial space and a more complex lost-like structure, which may contribute to the hepatic function.

Amino acid catabolism primarily occurs in the liver [18]. RNA-Seq results showed that the DEGs in this study mainly participated in the metabolism of amino acids and their derivatives. Though PKM only contains 14–21% crude protein [19], the amino acid utilization of PKM is high [20]. Except for valine and glycine, the digestibility of most amino acids is above 85% [21]. The higher digestibility of palm kernel meal was probably the primary reason the differential genes were mainly enriched in the amino acids and derivatives pathway.

**Fig. 9:**
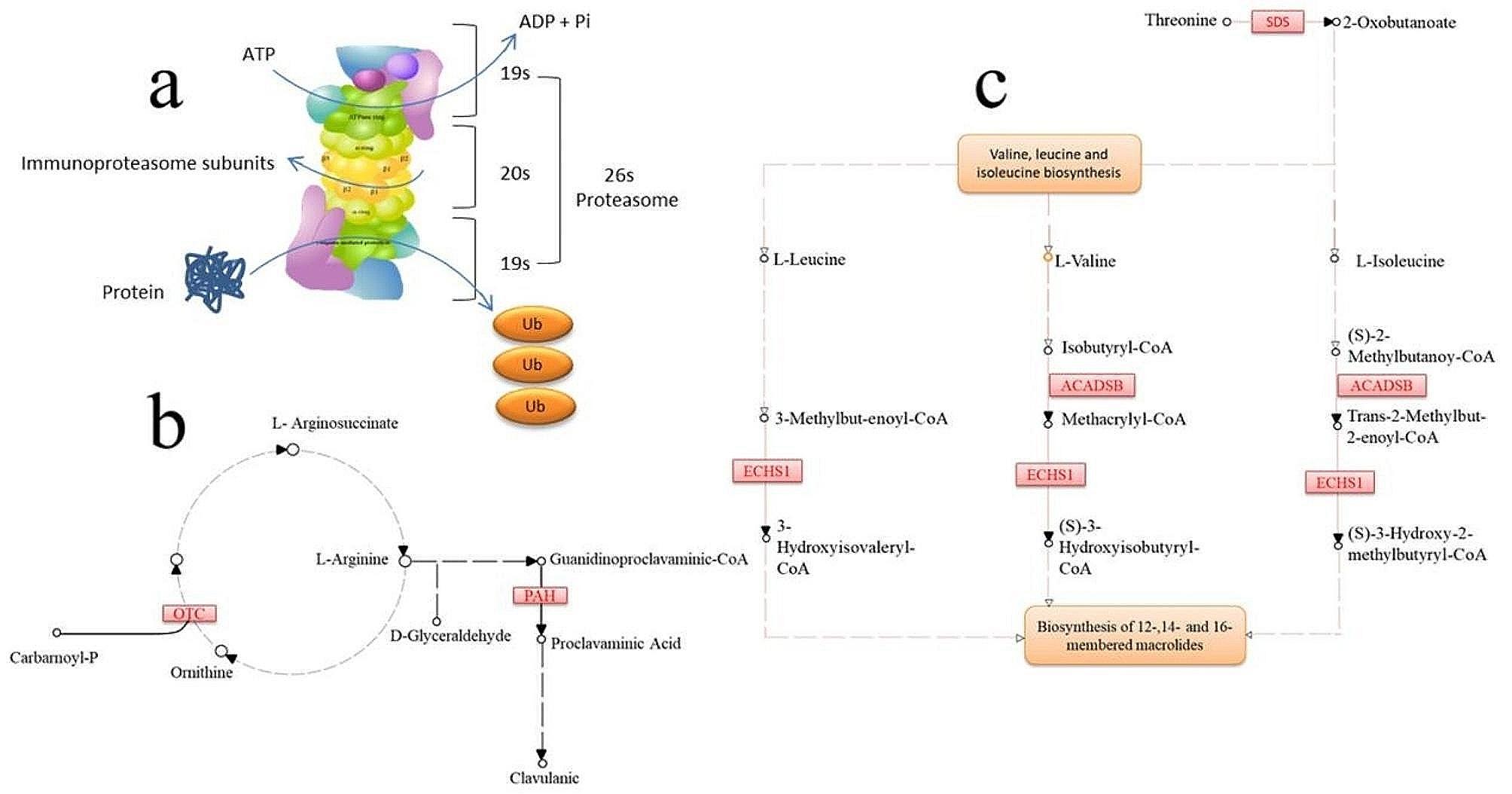
Metabolism of amino acids and derivatives. a: Ubiquitin-dependent protein degradation. b: Glycolysis/gluconeogenesis, glycine, serine, and threonine metabolism. c: Valine, leucine and isoleucine biosynthesis

Amino acids are used to synthesize proteins, peptides, and other nitrogen-containing substances uniquely required by the organism [22]. Amino acids are also degraded into α-keto acids [23], amines [24], and carbon dioxide through deamination, transamination [25], and a combination of deamination or decarboxylation. The α-keto acids can be converted into glucose, lipids, re-synthesized into some non-essential amino acids [26], or oxidized to carbon dioxide and water through the tricarboxylic acid cycle to release energy. *PSMC3*,* PSMC5*,* PSMD4*,* PSME1*,* ACADSB*,* ECHS1*,* SDS*,* OTC*, and *PAH* were the main DEGs in this study. These genes were categorized into three major groups comprising proteasome family genes (*PSMC3*,* PSMC5*,* PSMD4*,* PSME1*), acyl coactivator genes (*ACADSB*,* ECHS1*), and genes that code for enzymes that regulate the metabolism of specific amino acids (*SDS*,* OTC*, and *PAH*). Component of the 26 S proteasome (PSMC) [27] is a multiprotein complex involved in ubiquitin-dependent protein degradation (Fig. 9a). This complex cycle plays a key role in maintaining protein homeostasis by removing misfolded or damaged proteins. Proteasome 26 S Subunit Ubiquitin Receptor (PSMD4) acts as a ubiquitin receptor subunit by interacting with ubiquitin motifs, where it selects ubiquitin conjugates for destruction. Thus, PSMD4 displays a preference for polyubiquitin chains [28]. Protein degradation mediated by the proteasome is essential for protein homeostasis and is strongly dependent on proteasome activator subunit (*PSME*) genes [29]. The three families of genes play an important role in the tricarboxylic acid cycle of the amino acid metabolic pathway. The reason for the significant expression of ATP kinase genes may be because the organism fully absorbs protein in the PKM diet. In oxidative metabolic activity organisms, the tricarboxylic acid cycle in the mitochondria requires large amounts of ATP to accomplish the digestion and absorption of nutrients, resulting in the differential expression of genes at the molecular level.

Short/branched chain acyl-CoA dehydrogenase (ACADSB) plays an important role in L-isoleucine metabolism by catalyzing the dehydrogenation of 2-methylbutyryl-CoA [30], one of the components of the L-isoleucine catabolic pathway. The protein encoded by the enoyl-CoA Hydratase-Short Chain 1 (*ECHS1*) gene functions in the second step of the mitochondrial fatty acid beta-oxidation pathway [31], where it catalyzes the hydration of 2-trans-enoyl-coenzyme A (CoA) intermediates to L-3-hydroxyacyl-CoAs. These two genes *ACADSB* and *ECHS1* are lysosomes that participate in the conversion of α-ketoglutarate to CoA after the production of α-ketoglutarate from amino acids in the first step of amino acid catabolism (Fig. 9c). The products of these genes promote amino acid catabolism. The Serine Dehydratase (*SDS*) gene encodes one of three enzymes involved in metabolizing serine and glycine. L-serine dehydratase converts L-serine to pyruvate and ammonia. Serine Dehydratase (*SDS*) can also metabolize threonine to NH4^+^ and 2-ketobutyrate. Ornithine Transcarbamylase (*OTC*) encodes a mitochondrial matrix enzyme [32]. The protein is involved in the urea cycle, where it converts ammonia into urea for excretion. Phenylalanine Hydroxylase (*PHA*) encodes a member of the biopterin-dependent aromatic amino acid hydroxylase protein family [33]. The phenylalanine hydroxylase enzyme hydroxylates phenylalanine to tyrosine, which regulates phenylalanine catabolism (Fig. 9b). Diseases associated with PHA include Phenylketonuria and Hyperphenylalaninemia. Differential expression of the discussed genes indicated that palm kernel meal diets participate in the amino acid decarboxylation and deamidation processes, significantly impacting the activity of other amino acids. How palm kernel meal regulates the expression of these genes is currently unknown, and this requires further investigation. Understanding the mechanism by which PKM promotes amino acid metabolism in the liver will accelerate its utilization as a feed supplement.

## Conclusions

Supplementing 18% PKM in diet significantly enhanced the antioxidation and immune response capacity in Tibetan sheep by enhancing the functioning of hepatocytes. At the molecular level, PKM increased the expression of genes involved in the amino acid metabolism in the liver. This study had one main limitation, it did not investigate the mechanisms by which PKM regulates amino acid metabolism. Further studies are needed to explore the molecular mechanism by which PKM promotes amino acid metabolism.

## Data Availability

The datasets presented in this study can be found in online repositories. The names of the repository/repositories and accession number(s) can be found below: NCBI SRA(accession: PRJNA1066079) https://dataview.ncbi.nlm.nih.gov/object/PRJNA1066079.

## Abbreviations

PKC: Palm kernel cake
IgA: Immunoglobulin A
IgM: Immunoglobulin M
TNF-α: Tumor necrosis factor-α
ELISA: Enzyme-linked immunosorbent assay
IL-1β: Interleukin-1β
SOD: Superoxide dismutase
T-AOC: Total antioxidant capacity
GSH-Px: Glutathione peroxidase
STEM: Short time-series expression miner
DEGs: Differently expressed genes
PCA: Principal component analysis
ECHS1: Enoyl-CoA Hydratase-Short Chain 1
SDS: Serine Dehydratase
OTC: Ornithine Transcarbamylase
PHA: Phenylalanine Hydroxylase
